# Acetylcholinesterase Inhibition and Antioxidant Activity of DHA‐Disubstituted Phospholipids

**DOI:** 10.1002/mnfr.70095

**Published:** 2025-05-13

**Authors:** Ernestina Garcia‐Quinto, Sabrina Sollecito‐Rovella, Victor M. Amador‐Luna, Lidia Montero, Gloria Fernandez‐Lorente

**Affiliations:** ^1^ Laboratory of Microbiology and Food Biocatalysis Institute of Food Science Research (CIAL, CSIC‐UAM) Madrid Spain; ^2^ Laboratory of Foodomics Institute of Food Science Research Madrid Spain

**Keywords:** acetylcholinesterase (AChE) inhibition, antioxidant activity, DHA disubstituted phospholipids (1,2‐Di‐DHA‐PC), docosahexaenoic acid (DHA), neurodegenerative diseases

## Abstract

Docosahexaenoic acid (DHA) is an essential fatty acid for the central nervous system. It plays a crucial role in brain health and the prevention of neurodegenerative diseases, particularly in its phospholipid form, which has greater bioavailability. Previous studies, conducted by our group, enabled the enzymatic synthesis of pure disubstituted DHA phospholipids (1,2‐Di‐DHA‐PC). In the present study, the inhibitory activities of 1,2‐Di‐DHA‐PC on acetylcholinesterase (AChE) and its antioxidant capacity were evaluated. The results showed that 1,2‐Di‐DHA‐PC exhibited significant inhibition of the AChE enzyme. Moreover, 1,2‐Di‐DHA‐PC showed antioxidant capacity compared to ascorbic acid, a natural antioxidant par excellence. These findings highlight the therapeutic potential of 1,2‐Di‐DHA‐PC in the treatment of neurodegenerative diseases and its ability to offer protection against the lipid peroxidation of the neuronal aging process, one of the main drivers of neurodegeneration, suggesting the need for further studies to confirm its clinical applicability.

## Graphical abstract

1,2‐Di‐DHA‐PC phospholipids in nutraceutical form may cross the blood‐brain barrier and inhibit acetylcholinesterase (AChE) activity, with an efficacy similar to galantamine. This suggests their potential as neuroprotective agents in the treatment of Alzheimer's disease.

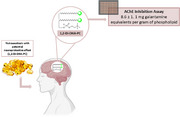

## 1 Introduction

Docosahexaenoic acid (DHA) is an important polyunsaturated fatty acid (PUFA) in the nervous system. It constitutes more than 40% of the PUFAs in neuronal and glial membranes. With aging and the development of neurodegenerative diseases, such as Alzheimer's disease (AD), a significant decrease in brain DHA has been observed [1], which affects to the membrane fluidity and neuronal homeostasis [2, 3].

Since there are no curative treatments for these diseases, prevention and risk reduction are crucial. Acetylcholinesterase (AChE) enzyme inhibitors have been shown to improve cognitive function by increasing acetylcholine levels in the brain, being relevant in the treatment of AD [4]. Currently, galantamine is the drug used to treat mild to moderate stages of AD. As an AChE inhibitor, galantamine has demonstrated efficacy in reducing the accumulation of beta‐amyloid (Aβ) plaques, which slows the progression of AD [5, 6].

Also, studies in animal models have shown that DHA plays a crucial role in protecting against the accumulation of Aβ. DHA administration has improved the levels of this fatty acid in the hippocampus and delayed the onset of Alzheimer's symptoms [1, 7]. These results suggest that DHA, administered as a nutraceutical, could complement the treatment of AD and allow a reduction in the dose of the drug, which, although it is one of the current treatments, generates side effects in patients. Galantamine side effects include symptoms such as nausea (24%), vomiting (14%), diarrhea (8%), abdominal discomfort, dyspepsia, anorexia, and weight loss (10%), all caused by cholinergic dependent activity [8].

Regarding the safety of DHA and EPA consumption, some studies have evaluated potential adverse effects, such as an increased risk of bleeding, alterations in glucose levels, or dysfunction of the immune system [9]. However, in Spain, according to the AESAN (Spanish Agency for Food Safety and Nutrition), the consumption of long‐chain omega‐3 fatty acids, such as DHA, is not associated with adverse effects in adults or children. The agency concludes that a combined supplemental intake of EPA and DHA of up to 5 g per day is safe for adults and does not increase these risks. In Europe, the intake of EPA and DHA through food and supplements is generally below 5 g per day; therefore, their consumption within these limits is considered safe for the adult population [10].

In recent years, it has been observed that the absorption of DHA in phospholipid form offers significant advantages in terms of bioavailability. This form allows DHA to pass intact through the gastrointestinal tract, reach the bloodstream as a sn‐2 lysophospholipid and cross the blood‐brain barrier (BBB) to integrate into neuronal membranes [11]. These phospholipids may offer additional benefits, such as antioxidant and anti‐inflammatory effects, which contribute to cognitive function [12, 13]. Therefore, they could play an important role in preventing or reducing the risk of developing neurodegenerative diseases [14, 15]. However, due to the low concentration of natural DHA in lipids, it is necessary to develop specific synthesis methods that allow higher concentrations to be obtained [16].

An effective strategy to address these nutritional needs is the synthesis of structured lipids or enzymatic modification of the position and composition of fatty acids in lipids [17]. Enzymes are catalysts of biological origin capable of catalyzing chemical processes with high catalytic activity, high specificity, and high selectivity [18]. In a recent study, García‐Quinto et al. addressed this challenge by enzymatic synthesis of phospholipids, using immobilized lipases and phospholipases for direct condensation of oleic acid and glycerophosphocholine. As a result, they were able to obtain disubstituted phospholipids of high purity, with a final yield of 78% [19]. Due to the good results obtained with oleic acid, it was decided to substitute it with DHA, given its greater relevance in the prevention of neurodegenerative diseases, thus achieving the synthesis of DHA‐structured phospholipids. Immobilized Quara LowP phospholipase was determined to be the optimal catalyst for the synthesis of DHA‐disubstituted phospholipids (1,2‐Di‐DHA‐PC), reaching a yield of 58% at 48 h [20]. Unlike conventional chemical catalysts, which require high temperatures, extreme conditions and generate toxic residues, enzymes are capable of catalyzing complex processes under mild conditions, leading to significant energy and economic savings [21].

In this work, the neuroprotective and antioxidant activities of our high purity DHA disubstituted phospholipids, enzymatically synthesized to ensure the incorporation of DHA at the sn‐2 position, which is the most bioavailable form according to the literature, are evaluated. For this purpose, in vitro assays of AChE enzyme inhibition and Oxygen Radical Absorbance Capacity (ORAC) are performed. Notably, this study represents the first evaluation of the neuroprotective and antioxidant activities of 1,2‐Di‐DHA‐PC, with no prior literature reporting such an analysis to date.

## 2 Materials and Methods

### 2.1 Materials

Docosahexaenoic acid (DHA) was obtained from the enzymatic hydrolysis of anchovy oil, which was purchased in capsules from NuaBiological at a Pharmacy (Madrid, Spain) and glycerophosphocholine (GPC) was purchased from Cayman Chemical Company (Michigan, USA). Soluble phospholipase Quara LowP (QlowP) was sent by Novozymes (Bagsvaerd, Denmark). Immobeads‐C18 IB‐ADS‐3 (C18) immobilization support was provided by ChiralVision (Leiden, The Netherlands) and 3Å pore size molecular sieve (2‐3 mm bead) was provided by Sigma‐Aldrich. Acetylcholinesterase (AChE) Type V1‐S from *Electrophorus electricus*, acetylthiocholine iodide (ACth), trizma hydrochloride (Tris‐HCl), phosphate‐buffered saline (PBS), ascorbic acid and fluorescein sodium salt. 4‐(amino‐359 sulfonyl)‐7‐fluoro‐2,1,3‐benzoxadiazole (ABD‐F), galantamine hydrobromide, and 2,2‐azobis(2‐aminodinopropane) dihydrochloride (AAPH) were purchased from TCI Chemicals (Tokyo, Japan). All the 96‐well microplate assays were performed in a spectrophotometer and fluorescence microplate reader (Cytation 5 imaging reader with auto‐disperser, BioTek Instruments, Winooski, VT, USA). 1,2‐didocosahexaenoyl‐sn‐glycero‐3‐phosphocholine (Di‐DHA‐PC) standard was purchased from Avanti Polar Lipids (Alabama, USA). All reagents and solvents used were of analytical grade.

### 2.2 Methods

#### 2.2.1 Enzymatic Synthesis of Structured Phospholipids From Docosahexaenoic Acid

**FIGURE 1:**
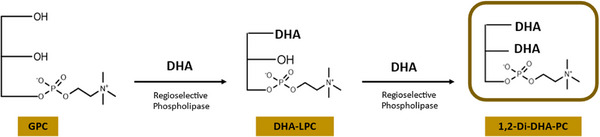
Schematic representation of the esterification reaction in an anhydrous medium, incorporating two docosahexaenoic acid (DHA) molecules onto a glycerophosphocholine (GPC) skeleton.

The esterification reaction with DHA was carried out in a solvent‐free, anhydrous medium, following the method described in [20]. The reaction mixture consisted of 250 mg of dry QlowP‐C18 immobilized phospholipase biocatalyst, 30 mg of GPC, 0.25 g of molecular sieve with a pore size of 3 Å, and 2.5 mL of free DHA, which was previously obtained through the enzymatic hydrolysis of anchovy oil. The reactions were conducted in glass vials at 60°C with constant stirring in an incubator at 150 rpm. Prior to adding the biocatalyst, the vials were incubated and stirred for 15 min to increase the solubility of GPC in the reaction medium. For each experiment, a negative control without the phospholipase derivative was performed under the same conditions. As illustrated in Figure 1, the enzymatic synthesis of structured DHA phospholipids catalyzed by immobilized phospholipase in an anhydrous medium is depicted.

The synthesis of disubstituted DHA phospholipids was monitored using HPLC, with samples taken from the supernatant. The experiments were performed in triplicate, and the standard deviation was always less than 5%. Garcia‐Quinto et al. demonstrated that the immobilized QlowP‐C18 phospholipase derivative was the most efficient catalyst, achieving a maximum 1,2‐Di‐DHA‐PC synthesis yield of 58% in 48 h, with a purity of 95% [20].

#### 2.2.2 Anti‐Cholinergic Activity

The anti‐cholinergic activity was measured by the inhibition of AChE enzyme with a fluorescent assay based on Ellman's method [22], with modifications described in Amador‐Luna et al. [23]. For the inhibition assay, a stock of the enzyme was prepared in 150 mM Tris‐HCl buffer at pH 8.00. In each well of the microplate, 100 µL of the same buffer, 25 µL of AChE enzyme (at a concentration of 0.8 U/mL) and different volumes (0–100 µL) of a solution of the pure phospholipid was added at an optimized concentration of 1000 µg/mL in EtOH/H_2_O (1:1, v/v). After 10 min of incubation, an auto‐dispenser attached to the microplate reader introduced 25 µL of ABD‐F (9.2 mM) in Tris‐HCl buffer at pH 8.00, and 50 µL of ACth was added and shaken to finally obtain the average speed of a kinetic measurement at *λ*_ex_ = 389 and *λ*_em_ = 513 nm for 15 min at the minimum possible interval at 37°C. Galantamine was used as a reference inhibitor following this protocol, and the result obtained for each sample was expressed as galantamine equivalents.

For all samples, the assay was performed twice. Each assay was performed in duplicate to ensure accuracy due to its high and precise repeatability. Specifically, for the disubstituted DHA phospholipid samples, two separate enzymatic reactions were performed.

#### 2.2.3 Antioxidant Activity

The oxygen radical absorbance capacity (ORAC) method was carried out to know the ROS scavenging capacity according to Ou et al. [24]. To obtain this value, 100 µL of extract at different concentrations in ethanol/water (1:9, v/v), 100 µL of AAPH (590 mM) in 30 mM phosphate‐buffered saline (PBS) at pH = 7.5, 25 µL of fluorescein (10 µM) in PBS buffer and 100 µL of PBS buffer were mixed in the microwell‐plate. Fluorescence was measured following the indications by Sánchez‐Martínez et al. [25]. Ascorbic acid was used as standard and the result obtained in each sample was given as equivalents of ascorbic acid. The assay was performed in duplicate.

## 3 Results and Discussion

### 3.1 AChE Inhibition

**FIGURE 2:**
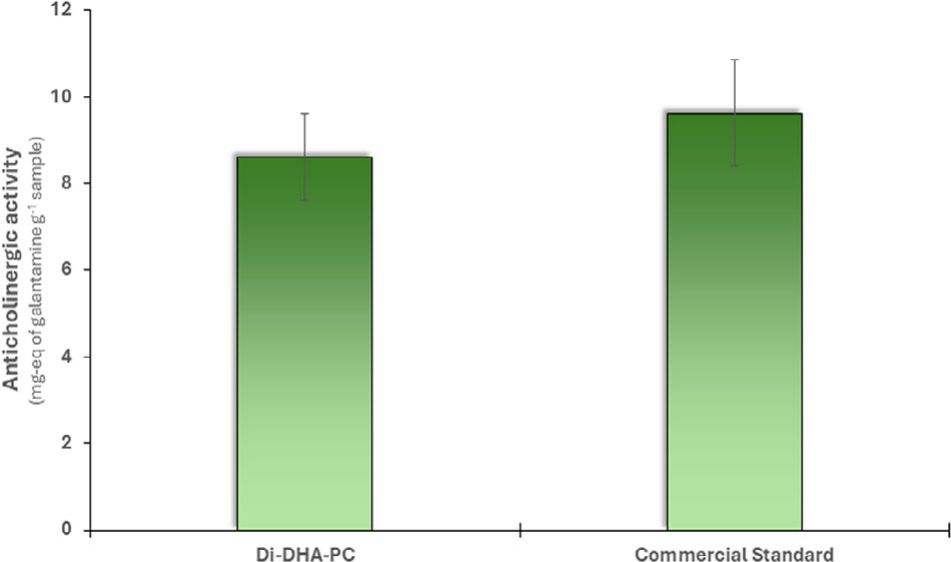
AChE inhibitory activity of enzymatically synthesized 1,2‐Di‐DHA‐PC compared to the commercial standard. The results are expressed in mg galantamine equivalents per gram of phospholipid. The results did not show significant differences compared to the commercial standard with a *p* value < 0.05.

To study the anticholinergic activity, the inhibitory capacity of the disubstituted DHA phospholipid (1,2‐Di‐DHA‐PC), enzymatically synthesized by our group, was evaluated on the AChE enzyme, using galantamine as a positive control due to its known efficacy in the inhibition of this enzyme in the treatment of AD [5, 6] and the result was compared with the inhibition exerted by the same chemically synthesized phospholipid and marketed by Avanti Polar Lipids (commercial standard). The results obtained for AChE inhibition are presented in Figure 2. The inhibitory activity of pure enzymatically synthesized Di‐DHA‐PC was quantified as 8.6 ± 1.1 mg galantamine equivalents per gram of phospholipid (IC50 of 118.8 ± 9.9 µg/mL), whereas the commercial standard showed an activity of 9.6 ± 1.2 mg galantamine equivalents per gram of phospholipid (IC50 of 107.1 ± 8.2 µg/mL). These results indicate that enzymatically synthesized 1,2‐Di‐DHA‐PC maintains a relevant inhibitory capacity, which do not present significant differences with the commercial standard, suggesting that the enzymatic synthesis process adequately preserves the activity of the compound. This finding highlights the potential of enzymes in the food industry as an outstanding alternative to traditional chemical methods for the synthesis of new functional ingredients with potential nutraceutical applications.

In light of these results, the AChE inhibitory capacity of enzymatically synthesized 1,2‐Di‐DHA‐PC is promising. So far, no similar studies comparing the neuroprotective activity of entirely pure DHA phospholipid samples have been found. However, studies on the anticholinesterase activity of different oil extracts containing a mixture of lipids, including omega‐3 PUFAs, but not exclusively DHA, do exist in the literature. Limanjaya et al. showed that tempeh oil, a traditional Indonesian fermented food made from white soybeans, at a concentration of 25 µg/mL, has the highest AChE inhibitory activity and does not differ significantly from soybean oil at the same concentration. As for the PUFAs present, alpha‐linolenic acid of the omega‐3 series was identified in both oils; however, the major component was predominantly omega‐6 [26].

On the other hand, providing DHA in the form of phospholipid could be one of the most efficient ways to transport this fatty acid to the brain, since phosphatidylcholine (PC) is one of the preferred vehicles to cross the BBB, which is relevant given the known neuroprotective effect of DHA [11]. In a recent publication, the inhibition of AChE was studied in two brown algae extracts, Lobophora Tsengii and Lobophora Australis [27]. In both extracts, phosphatidylcholine and phosphatidylethanolamine were identified as the main components of phospholipids, in which polar lipids were predominant in relation to total lipids (43.47% in *L. tsengii* and 48.95% in *L. australis*). These phospholipids contributed largely to a strong inhibition of AChE, as does our 1,2‐Di‐DHA‐PC phospholipid in this study. Nevertheless, the purely synthesized 1,2‐Di‐DHA‐PC might offer additional therapeutic benefits compared to extracts containing various lipids, especially in the treatment of neurodegenerative diseases such as AD.

Finally, since galantamine is administered in daily doses of 8, 16 and 24 mg to treat AD, adjusted according to the severity (mild or moderate) [28], and considering that our phospholipid 1,2‐Di‐DHA‐PC has an inhibitory activity equivalent to 8.6 ± 1.1 mg of galantamine per gram, 1 g of 1,2‐Di‐DHA‐PC could provide an equivalent dose for mild stages of the disease. This suggests that administration of the enzymatically synthesized phospholipid could allow a reduction in the dose of galantamine, thereby mitigating its common side effects, such as nausea, vomiting, diarrhea, and so forth [8]. Moreover, galantamine is contraindicated in patients with significant renal or hepatic impairment, highlighting the need for alternative or complementary therapeutic approaches [29].

According to the EFSA and AESAN, DHA and its derivatives are considered safe at doses up to 5 g/day, though amounts exceeding 3 g/day may cause mild gastrointestinal discomfort [10]. Given that the proposed 1 g/day dose is well below this threshold, it presents a favorable safety profile. Furthermore, 1,2‐Di‐DHA‐PC could enhance DHA bioavailability and facilitate its targeted delivery to the brain, thereby strengthening its neuroprotective potential in Alzheimer's disease.

### 3.2 Antioxidant Activity

**FIGURE 3:**
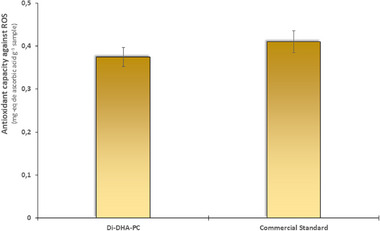
Antioxidant activity of enzymatically synthesized 1,2‐Di‐DHA‐PC compared to the commercial standard. The results are expressed in mg equivalent of ascorbic acid per gram of sample. The results did not show significant differences compared to the commercial standard with a *p* value < 0.05.

The antioxidant activity of the disubstituted DHA phospholipid (1,2‐Di‐DHA‐PC) was evaluated by the ORAC method, which measures the ability of antioxidants to neutralize peroxyl free radicals generated by the AAPH agent. Ascorbic acid was used as a positive control due to its recognized high antioxidant activity. The results obtained for antioxidant activity are shown in Figure 3.

The enzymatically synthesized 1,2‐Di‐DHA‐PC showed an antioxidant activity of 0.37 ± 0.02 mg ascorbic acid equivalent per gram, while the commercial standard 1,2‐Di‐DHA‐PC exhibited an activity of 0.41 ± 0.03 mg ascorbic acid equivalent per gram. Although ascorbic acid is significantly stronger, both enzymatically synthesized 1,2‐Di‐DHA‐PC and the commercial standard exhibited considerable antioxidant capacity, with values close to each other. These results suggest that 1,2‐Di‐DHA‐PC maintains a relevant antioxidant activity, similar to that of the commercial standard, indicating that enzymatic synthesis does not adversely affect its antioxidant properties. Knowing the remarkable advantages of enzymes and given that long‐chain ω‐3 PUFAs are very sensitive to oxidation and process conditions, enzymatic methods are definitely more suitable than chemical ones, especially when specific localization of the incorporated fatty acids is desired [30, 31].

These results are consistent with previous studies that have reported similar antioxidant capacities for other compounds and natural extracts. For example, a study of *Dunaliella salina* extracts obtained by conventional supercritical extraction revealed an antioxidant activity of 0.3 mg ascorbic acid equivalent per gram of extract [23]. The β‐carotene, the predominant carotenoid in this microalgae, has demonstrated a high antioxidant capacity against oxygen radicals [32]. Comparable to β‐carotene in *D. salina* extract, 1,2‐Di‐DHA‐PC exhibits antioxidant activity, indicating its potential suitability for applications requiring an antioxidant profile. The antioxidant efficacy of 1,2‐Di‐DHA‐PC, with the amphipathic character of phosphatidylcholine allowing it to easily cross the BBB, reinforces its potential in contexts where protection against oxidative damage is crucial, such as in neurodegenerative diseases.

## 4 Conclusions

A gram of 1,2‐Di‐DHA‐PC, a disubstituted DHA phospholipid made exclusively by enzyme synthesis, demonstrated an impressive inhibitory power against acetylcholinesterase (AChE) of 8.6 ± 1.1 mg galantamine equivalents. Its extratherapeutic potential for the treatment of neurodegenerative diseases is highlighted by its capacity to inhibit AChE and its suggested function in DHA transport to the brain, as reported in the literature. 1,2‐Di‐DHA‐PC could facilitate a reduction in the dose of galantamine required, which could minimize the side effects associated with this drug. To the best of our knowledge, this work reports the first results on AChE inhibition of DHA‐disubstituted phospholipids, and the results demonstrate positive anticholinergic action. In light of these unexpected findings, more research on the bioavailability, safety, and clinical efficacy of Di‐DHA‐PC would be required to confirm its value as an adjuvant in the treatment of AD.

The enzymatically synthesized 1,2‐Di‐DHA‐PC was found to have an antioxidant capacity of 0.37 ± 0.02 mg ascorbic acid equivalents per gram, as determined by the ORAC method. These results underline the potential of 1,2‐Di‐DHA‐PC for applications requiring protection against oxidative damage. The combination of providing DHA as a phospholipid together with its antioxidant capacity suggests benefits in therapeutic contexts related to neurodegenerative diseases. However, further studies are needed to explore its efficacy and applicability in these areas.

## Conflicts of Interest

The authors declare the following financial interests/personal relationships which may be considered as potential competing interests: Gloria Fernandez‐Lorente reports financial support was provided by Spanish Scientific Research Council. Gloria Fernandez‐Lorente reports a relationship with Spanish Scientific Research Council that includes: employment.

## Data Availability

The authors are unable or have chosen not to specify which data has been used.
